# Translational Considerations for Injectable Biomaterials and Bioscaffolds to Repair and Regenerate Brain Tissue

**DOI:** 10.1002/adhm.202501711

**Published:** 2025-09-15

**Authors:** Michel Modo, Alena Kisel

**Affiliations:** ^1^ Department of Radiology University of Pittsburgh Pittsburgh PA 15203 USA; ^2^ Department of Bioengineering University of Pittsburgh Pittsburgh PA 15203 USA; ^3^ McGowan Institute for Regenerative Medicine University of Pittsburgh Pittsburgh PA 15203 USA; ^4^ Centre for Neuroscience University of Pittsburgh Pittsburgh PA 15203 USA

**Keywords:** biomaterial, bioscaffold, clinical translation, extracellular matrix, glioma, hydrogel, regeneration, repair, resection, stroke, traumatic brain injury

## Abstract

Adult neurogenesis can replace lost neurons through migration and participate in the repair of damaged tissues. Neurogenesis by itself is known to be insufficient to replace lost tissue. Injectable bioscaffolds derived from extracellular matrix (ECM) have shown promise in repairing and regenerating brain tissue. These bioscaffolds need to be considered within their pathological context (e.g. proteases), which contribute to its biodegradation. Biodegradation through peripheral immune cells is required to promote the invasion of brain cells and reconstitute a *de novo* tissue. In addition to the biomaterial characteristics, a greater focus on translational considerations (e.g., intracerebral delivery) is required to establish a robust pathway to the clinic. Especially advances in developing large animal models will be required to address key issues, such as regrowing of a gyrencephalic brain, as well as potential limitations to tissue regeneration due to the size of the volumetric deficits. It is advocated that non‐human primates will be an essential step prior to first‐in‐human investigations. Injectable bioscaffolds have the potential to promote a paradigm shift in the treatment of acute brain injuries, but this can only be achieved through a robust and potentially iterative translational effort.

## Graphical abstract

The repair and regeneration of brain tissue faces both biological and technical challenges. Injectable bioscaffolds offer new opportunities to stimulate tissue regrowth in the brain by recruiting neural stem cells. Here, the translational issues are reviewed that need to be address to advance this promising new therapeutic approach from the bench to the bedside.

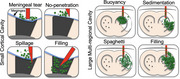

## 1 Introduction

The repair and regeneration of the brain poses a multitude of biological and technical challenges.^[^ ^1^ ^]^ Unlike other organs, such as the liver and skin, the brain does not regenerate tissue spontaneously, and it only has a limited capacity to repair damaged tissues.^[^ ^2^ ^]^ Both processes are dependent on the availability of neural stem cells (NSCs), which are fundamental to building brain tissue.^[^ ^3^ ^]^ Two main endogenous neurogenic zones, notably the subventricular zone (SVZ) along the lateral ventricle and granule cell layer of the dentate gyrus (DG) in the hippocampus, exist in humans, and these niches persist into adulthood.^[^ ^4^ ^]^ During tissue homeostasis, there is a low turnover of cells within the adult human brain,^[^ ^5^ ^]^ but tissue damage induces a dramatic increase in neurogenesis that is inflammation‐mediated and aimed at tissue repair.^[^ ^6^ ^]^

An upregulation of neurogenesis in the SVZ has been documented in response to an acute brain injury, such as stroke and traumatic brain injury, in human patients,^[^ ^7^ ^]^ but neurogenesis under non‐pathological conditions in the adult human DG appears to be very low.^[^ ^5^, ^8^ ^]^ Neurogenesis along the SVZ reflects a positional specification that replicates brain development.^[^ ^3^ ^]^ Adult neurogenesis hence, maintains the ability to replace lost neurons in striatal and cortical tissues through the migration of tissue‐specific neural progenitor cells (NPCs). Supplementation of this endogenous repair response can be achieved by implantation of NSCs into the damaged tissue.^[^ ^9^ ^]^ These NSCs/NPCs participate in tissue repair, but by themselves do not regenerate lost tissue.^[^ ^10^ ^]^ Tissue regeneration here refers to the re‐establishment of a *de novo* tissue. After an acute brain injury, a chronic tissue cavity remains at the site of the lesion core.^[^ ^11^ ^]^

These cavities fill with extracellular fluid (ECF) and are void of cells, extracellular matrix, or vasculature. Hence, there is no structural support within this cyst to support the invasion of cells from the surrounding brain tissue. Implanted cells also lack the structural support to repopulate the cavity.^[^ ^9^ ^]^ However, implantation of NSCs with microstructural support^[^ ^12^ ^]^ or embedded within hydrogels can repopulate this cavity with cells.^[^ ^13^ ^]^ To ensure the long‐term survival of newly forming tissue within the cavity, a neovasculature needs to be established by stimulating an ingrowth of host vasculature using growth factors, such as vascular endothelial growth factor‐A (VEGF‐A),^[^ ^12^, ^14^ ^]^ or by co‐implanting endothelial cells (ECs) that organize into a vascular bed that meets the *de novo* tissue's metabolic demands.^[^ ^15^ ^]^ Endothelial morphogenesis is highly influenced by neural cells, and both are required to deposit extracellular matrix (ECM) to form a *de novo* tissue with a blood‐brain barrier (BBB).^[^ ^16^ ^]^ A major downside of allogenic endothelial cells is their high immunogenicity, and hence recruitment of an endogenous cell‐based vasculature is advantageous. Bioscaffolds formed out of ECM contain inductive cues, such as VEGF‐A,^[^ ^17^ ^]^ that recruit endogenous neural and vascular cells into the tissue cavity.^[^ ^18^ ^]^ These inductive bioscaffolds promote the gradual reconstitution of brain tissue without requiring implantation of any cells.^[^ ^19^ ^]^

Inducing an endogenous regenerative process is potentially advantageous, as no exogenous cells need to be transplanted. In peripheral tissues (e.g., muscle and heart), inductive acellular bioscaffolds are sufficient to promote endogenous tissue regeneration. Decellularization of tissues affords the isolation of ECM, including trapped growth factors and matrix‐bound nanovesicles (MBVs), but all cellular debris (e.g., DNA) is being removed. ECM‐based bioscaffolds can be formulated as sheets to, for instance, stimulate skin regeneration, but can also be prepared as hydrogels that can fill volumetric tissue defects, as in the case of volumetric muscle loss or brain cavities.^[^ ^19^, ^20^ ^]^ In addition to these natural bioscaffolds, bottom‐up engineered hydrogels that commonly incorporate only a selective set of ECM molecules (e.g., hyaluronic acid) or growth factors (e.g., VEGF‐A, EGF) are also being evaluated for brain tissue regeneration.^[^ ^21^ ^]^ These injectable bioscaffolds offer a promising approach to treat acute brain injuries by providing a supportive microenvironment for cell growth, differentiation, and tissue regeneration.^[^ ^2^ ^]^ This review explores the translational considerations of injectable bioscaffolds for brain tissue repair and regeneration.

## 2 Interventional Access to Brain Tissue

Unlike most soft tissues, the brain is protected by the skull. Intervening in the brain, hence, requires access routes. Although intra‐nasal and intra‐vascular strategies exist and have been exploited to deliver pharmaceuticals and biologics to the brain, delivery of large volumes of bioscaffolds into a lesion cavity is challenging using these routes. Endovascular delivery through catheters can improve targeting of a particular brain region or tumor.^[^ ^22^ ^]^ Endovascular mechanical thrombectomy is increasingly used to remove blood clots and manage aneurysm in children and adults,^[^ ^23^ ^]^ as it is less invasive than open skull surgery. Direct access to damaged tissue using this minimally invasive procedure would be advantageous for patient management, but it currently remains unclear if large quantities of biomaterials could be delivered using this method and might require the development of novel endovascular probes.

### 2.1 Transcranial Access

**Figure 1:**
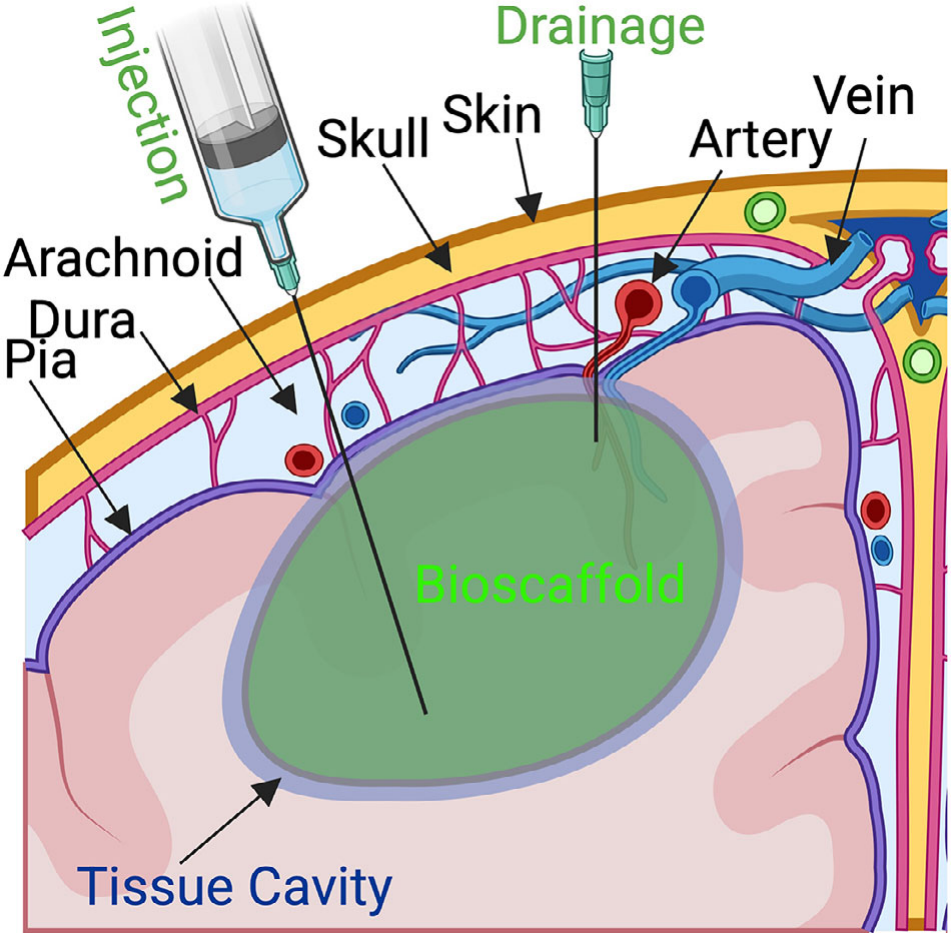
Intracerebral access for bioscaffold delivery. Illustration of the different tissue layers that need to be traversed to access the brain using a transcranial approach. Each tissue layer will need to be reconstructed to ensure adequate protection of brain tissue.

Transcranial access to brain tissue is limited by the skull, but a penetration through the overlying tissues, commonly referred to as the meninges, is also required (**Figure** **1**). Neurosurgeons, therefore, have to create access points by either drilling a small burr hole (< 1 cm in diameter) into the skull in a procedure known as trepanation (a.k.a. trephination, trephining, and trepanning) or the surgical removal of a larger piece of skull.^[^ ^24^ ^]^ If the skull piece or an artificial replacement (e.g., titanium plates) is repositioned immediately after the surgical procedure is complete, the procedure is referred to as a craniotomy (e.g., tumor removal), whereas a replacement through a delayed secondary surgical procedure is known as a craniectomy (e.g., decompression surgery).

### 2.2 Cranioplasty

Cranioplasty is the surgical repair of the rigid bone tissue that constitutes the skull. A variety of clinical products are available.^[^ ^25^ ^]^ Smaller skull defects, such as those involving burr holes, can be concealed using a polymeric covering device^[^ ^26^ ^]^ or filled using hemostatic materials that can promote the regrowth of the bone.^[^ ^25a^ ^]^ Bone wax, which is composed of beeswax and a softening agent, has been used for over a century.^[^ ^27^ ^]^ It stops bleeding from the surrounding bone, while providing a physical barrier between the outside and inside of the skull. It is malleable to the defect in situ, hence providing an easy‐to‐use material to repair small skull defects.^[^ ^28^ ^]^ In animals undergoing stereotactic procedures, bone wax is commonly used to fill the bone defect. An alternative to bone wax consists of “bone dust”.^[^ ^29^ ^]^ Bone dust is a side‐product of trephination and can be collected during this procedure. Mixture with fibrin glue renders it malleable with filling properties that can reconstitute the skull bone.^[^ ^30^ ^]^ However, insufficient bone dust might be collected, and hence, allogenic or synthetic material is increasingly being investigated for cranioplasty. Bioprinting of bioresorbable scaffolds for bone tissue engineering is achieving promising results and constitutes state‐of‐the‐art burr hole repair.^[^ ^31^ ^]^ In addition to burr holes, skull flaps, and repositioning also requires material to fill in bone gaps. Hydroxyapatite, acrylic resins, polyethylene, or ceramic materials have been used for this purpose.^[^ ^25^, ^32^ ^]^

### 2.3 Duraplasty

In addition to creating an access through the skull, neurosurgeons need to resect 3 membrane layers referred to as meninges covering the brain. These each have unique biological features and functions. The meninges consist of the dura mater just below the skull, the arachnoid mater, and directly over the brain lies the pia mater.^[^ ^33^ ^]^ The arachnoid and pia mater are also sometimes collectively termed the leptomeninges (i.e., thin meninges). The dura consists of two thin cell layers, with the periosteal layer being the outermost component. This layer has interleaved cellular processes that are highly fibroelastic and does not contain extracellular collagen. The endosteal layer underneath (also sometimes referred to as dural border cell layer or meningeal dura mater) is a dense fibrous tissue composed of “flattened” fibroblasts producing extracellular collagens. Both layers are separated at the dural venous sinuses, a major site of blood efflux from the brain. The dura is essential for the reabsorption of CSF and the venous return of cerebral blood. The arachnoid mater (the arachnoid in short) derives its name from a spider‐web‐like appearance. It is physically detached from the dura, but has small protrusions known as arachnoid villi communicating with the venous sinuses. It does not penetrate sulci. Between the arachnoid and pia mater lies the subarachnoid space, a meshwork of trabeculae, which is filled with CSF. The pia mater is derived from the embryonic mesoderm and consists of a thin fibrous tissue that traces the surfaces of brain tissue, permeable to small solutes and CSF. Brain vasculature crosses this membrane and is considered part of the glymphatic drainage system. It is anchored to the brain through the endfeet of astrocytes. A physiological integration of *de novo* neocortical brain tissue with the arachnoid is hence required to restore a functional glymphatic system.

Craniotomy typically leads to a major disruption or resection of the meninges, whereas a burr hole leads to a smaller disruption, typically from a cannula being introduced. Repairing or promoting the regeneration of these meninges is essential to ensure protection of the brain, as well as to support the surface of the brain's physiological environment. To prevent CSF leakage and maintain the immunoprivilege of the brain, damage or disruption of the meninges needs to be repaired prior to bone repair. During craniotomy, the delicate resection and preservation of meninges affords autologous grafting, where the meninges are re‐positioned and sutured in place. Similarly, allo‐ and xenografts (especially porcine) material is available to reseal the dura. Classical patching of the meninges was achieved through bioadhesives, such as fibrin (e.g., Tiseel, Artiss) or fibrinogen‐thrombin composites.^[^ ^34^ ^]^ Natural dura substitutes are typically collagen‐based sheets (e.g., DuraGen, DuraForm, Tutopatch, and AlloDerm) that can be placed on top of the brain. Synthetic alternatives (e.g., Synthecel, Cerafix, and DuraSeal) are increasingly evaluated to replace the dura and provide stronger sealing properties than natural materials.^[^ ^35^ ^]^ Duraplasty refers to the surgical procedure used to repair or replace the dura mater.^[^ ^36^ ^]^

Instead of merely resealing the dura, some clinical products formulated as sheets of collagen are thought to promote the regeneration of the dura and meninges from surrounding host tissue. These inductive natural materials (i.e., collagen) undergo biodegradation by the host immune system. However, the focus is often solely on resealing the dura, rather than repairing or replacing the arachnoid and pia. Sponge‐like collagen sheets combined with coagulants like fibrinogen (e.g., TachoSil) have also been used and resemble more the arachnoid than the dura. Layered combinations of the materials can be conceived specifically designed for meninges regeneration/repair. It remains unclear if inductive collagen‐based layered sheets of different formulation (e.g., sponge‐like layer replicating arachnoid properties, plus a dense sealant layer mimicking the dura) could re‐establish the 3‐layer configuration of meninges, hence providing a more physiological restoration compared to synthetic materials. Burr hole surgery typically just creates a small tear, with dura tenting sutures being sufficient to re‐establish layer integrity.^[^ ^37^ ^]^ In small animals undergoing stereotactic injections, typically no repair of the meninges is undertaken, as it is expected that these spontaneously restore their physiological environment. Beveled needles/cannulae are advantageous, as these can cut just a small tear in the meninges to access brain tissue, whereas blunt needles/cannulae can tear larger areas of the meninges and create a major neuroinflammatory event.

### 2.4 Minimally‐Invasive Image‐Guided Bioscaffold Delivery

**Figure 2:**
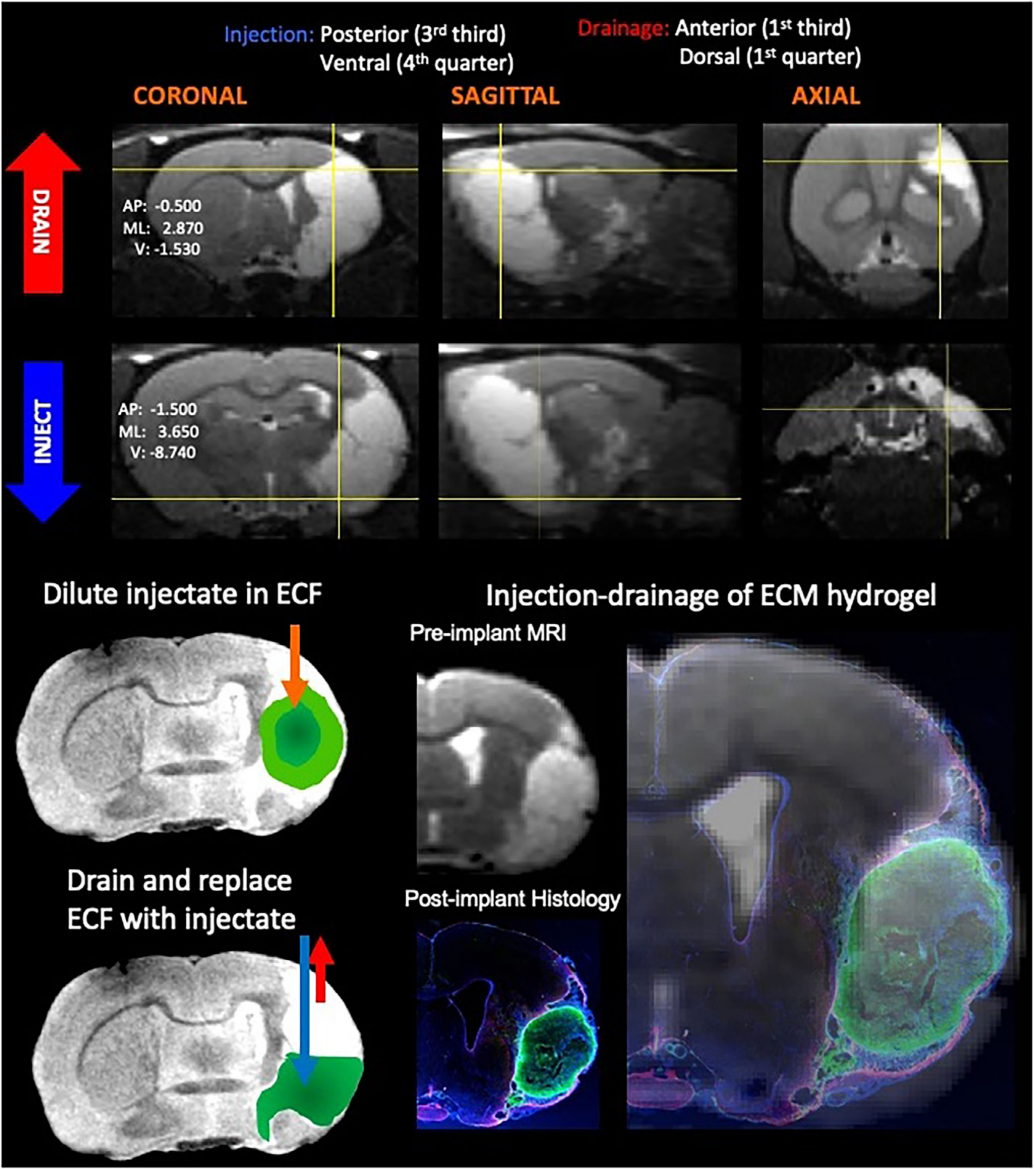
Image‐guided bioscaffold delivery. Magnetic resonance imaging (MRI) is used to measure the cavity volume, as well as to define stereotactic coordinates for the positioning of injection and drainage cannulae.^[^ ^12b^ ^]^ In case of solid bioscaffolds, such as microparticles, these need to be injected into the extracellular fluid (ECF) filling the cavity and diluted in this liquid. In contrast, hydrogels can be injected to displace the ECF, while this is being drained through a second cannulae. This injection‐drainage filling of, for instance, the stroke cavity, completely fills the tissue void with the bioscaffold.^[^ ^38^ ^]^

**Figure 3:**
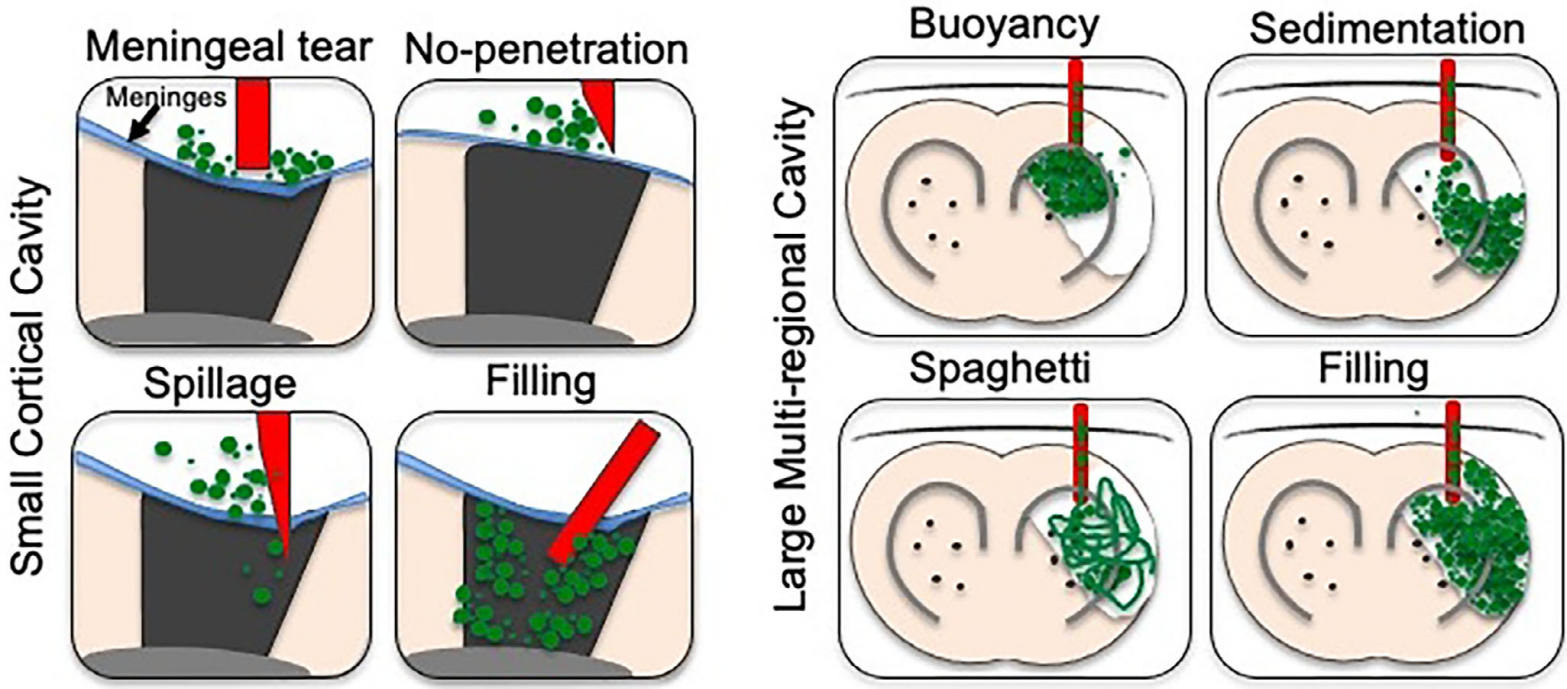
Intracerebral delivery failures and success. The implantation of injectable bioscaffolds is highly dependent on the nature, the location and volume of tissue loss. A small cortical tissue cavity is best filled by angulating a beveled injection needle to cut a small hole through the meninges and deliver the bioscaffold below these brain‐encapsulating layers.^[^ ^15a^ ^]^ Fluid from the cyst evacuates along the needle. In contrast, deep seated large volume lesion cavities are best filled using an injection‐drainage approach. The material density is an important factor to avoid buoyancy. In case of microparticles or organoids the sedimentation is an issue if these are not suspended in a hydrogel. Shear stress and ejection pressure, as well as in syringe/needle cross‐linking, can also modify the bioscaffold structures and result in for instance spaghetti‐like strings being injected rather than a volume‐filling liquid.

**Figure 4:**
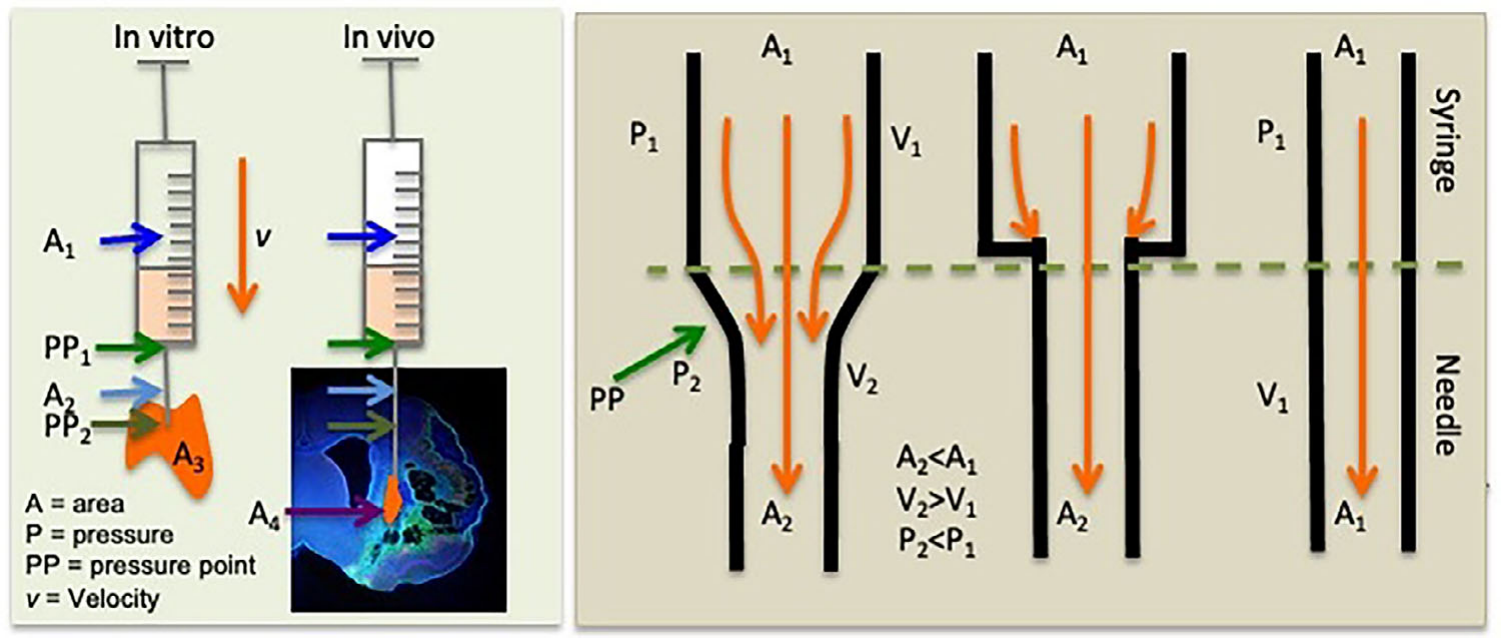
Injection/Ejection‐induced shear stress. Conceptualization of cell and bioscaffold ejection through a syringe/needle combination.^[^ ^39^ ^]^ In vitro*/*ex vivo ejections model the pressure and afford measurements in the ejectate to determine how ejections affect these materials. However, in vivo the presence of tissue or liquid will affect these measurement (e.g., increased pressure required to inject into a tissue than air). The type of barrel connection between the syringe and needle/cannulae can create pressure points that change the shear stress and pressure within each compartment. A single barrel syringe/needle device is preferable, as this has no pressure point or connection that could increase shear stress.

The most common delivery of bioscaffolds into brain tissue is likely a minimally invasive approach through a burr hole in the skull. This necessitates a thin needle that can access deeply seated tissues or cavities by traversing overlying tissue, whilst minimizing the damage caused to this “intact” brain tissue. To inform the volume and site of delivery, non‐invasive imaging is required (**Figure** **2**). For instance, magnetic resonance imaging (MRI) can be used to measure the volume of a tissue cavity, as well as provide surgical planning for the placement of cannulae.^[^ ^12^, ^38^ ^]^ To avoid changes in intracranial pressure, ECF filling the cavity can be evacuated through a secondary drainage cannulae.^[^ ^38^ ^]^ Cannulae/needles need to be lowered slowly through brain tissue to avoid tissue tearing and deformation. Beveled cannulae are preferable to minimize the disturbance of the axons in the corpus callosum. Placement of bioscaffolds or tissue constructs in the neocortex is potentially more challenging without overlying tissues retaining the injectate. In these cases, angulated injections of bioscaffolds under the meninges, without direct drainage through a secondary cannulae, might be more efficient^[^ ^15a^ ^]^ (**Figure** **3**). Shear stress of delivering materials through a thin needle needs to be considered in this process,^[^ ^39^ ^]^ as it can modify the ejectate (**Figure** **4**). Inclusion of cells needs more extensive consideration than bioscaffolds alone. Notably, the suspension density, as well as the viability of cells, are a concern in a translational setting, but no clear limits for these have been established.^[^ ^40^ ^]^ Delivery of *ex vivo* tissue constructs or organoids also requires consideration of their configuration. A larger construct size is more challenging to deliver viably through a thin needle than cell suspensions.^[^ ^15^, ^41^ ^]^ The delivery of injectable bioscaffolds to the brain hence, poses a unique opportunity that can be applied to most cases of acute brain injuries, such as stroke and traumatic brain injury, but also faces significant technical constraints.

## 3 Design Considerations for Brain‐Injectable Bioscaffolds

### 3.1 Injectable Hydrogels

Injectable bioscaffolds need to pass through a needle for delivery. To be injectable, these materials must either be in a liquid or slightly viscous phase to physically conform to the syringe and needle barrel. Solid bioscaffolds that are designed as micro‐ or nanoparticles can also be injected, but typically need to be suspended in a liquid medium for delivery (e.g., a sol or sol–gel preparation). The viscosity of the bioscaffolds is hence a major determinant of their injectability. For instance, urinary bladder matrix (UBM)‐based ECM hydrogels at lower concentrations (< 4 mg mL^−1^) have a low viscosity (≈0.05 Pa*s), but higher protein concentration (> 6 mg mL^−1^) have much higher viscosities (> 0.2 mg mL^−1^).^[^ ^42^ ^]^ Higher viscosities lead to more difficult uptake into the syringe, and a higher force is needed for ejection (i.e., increasing ejection pressure). Use of larger diameter needles with higher viscosity materials can reduce some of these issues, but these will lead to more tissue damage while traversing brain tissue.^[^ ^40a^ ^]^ Shear stress during the ejection process can affect protein structure, as well as cross‐linking of proteins, and consequently change the material properties of the injected bioscaffold. Inclusion of microparticles, cells, tissue constructs, or organoids will affect the viscosity of the material depending on their volume fraction,^[^ ^15^, ^40^ ^]^ but these materials are also more vulnerable to shear stress and can potentially lead to cell death or phenotypic changes.^[^ ^39^ ^]^ Viscosity, needle/syringe barrel size, and the resulting shear stress exposure during ejection are hence important factors to consider in choosing an appropriate bioscaffolding material. No clear guidance is currently available as to what conditions are limiting potential therapeutic applications. Some aspects might be challenging in small rodents (i.e., 20G needle size), but might be less of a concern in larger species, such as humans.

**Figure 5:**
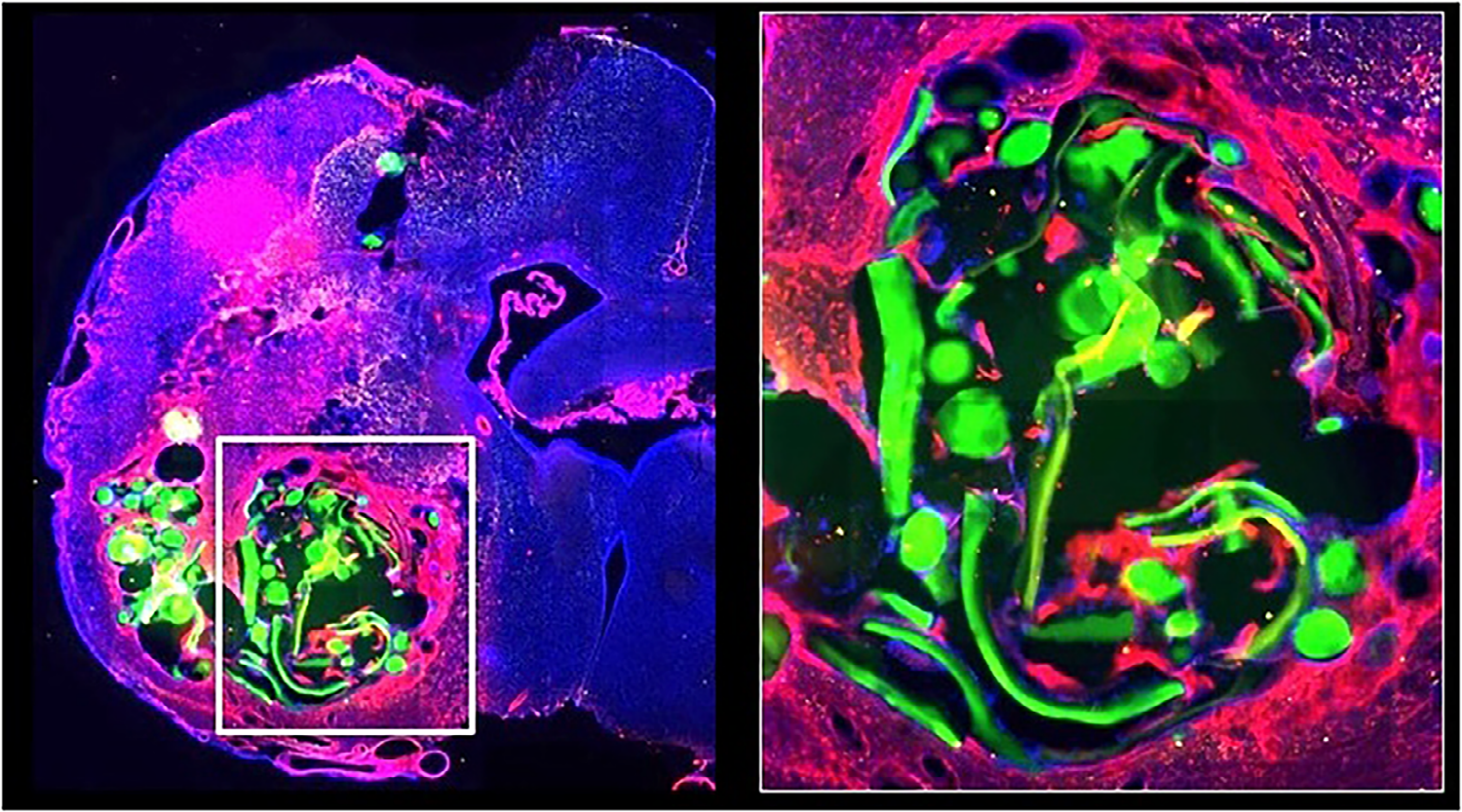
Shear stress‐induced bioscaffold deformation. Example of Alexa Fluor 488‐containing polyethylene glycol (PEG) microspheres (in green) implanted within UBM‐ECM‐based hydrogel.^[^ ^15a^ ^]^ Shear stress during the delivery process re‐configured the microsphere material as strings that looked like spaghetti after injection. These observations indicate that a re‐design of the delivery process needs to be undertaken.

In addition to material viscosity, the time to gelation of a bioscaffold is an important variable in a translational setting. Preparation of the bioscaffold prior to injection requires it to be maintained in a liquid form. In the case of hydrogel, this pre‐gel preparation needs to be loaded into the syringe, mounted on a stereotactic frame, and injected. Time‐to‐gelation determines when the pre‐gel is no longer in an injectable form that would adapt to the cavity topology. UBM‐ECM hydrogels (3–8 mg mL^−1^ preparations) achieve gelation in ≈10 min at body temperature, hence providing a narrow time frame during which these scaffolds can be injected.^[^ ^42^ ^]^ At a rate of 10 µL min^−1^, a 100 µL cavity requires 10 min of continuous pre‐gel injection.^[^ ^38^ ^]^ Cooling of syringes prior to injection can extend this time window, with gelation only being initiated at body temperature after injection. Leaving the needle in place for 10 min after injection ensures gelation occurred and that there is minimal reflux of pre‐gel. If gelation is initiated in the syringe, gelation can occur and lead to the extrusion of “spaghetti” that will fill up the cavity (**Figure** **5**). However, this does not provide a snug interface with brain tissue to promote tissue regeneration. Conversely, if time‐to‐gelation is too long, an injection‐drainage approach risks washing‐out of the injected material. The use of light‐induced cross‐linking reagents can achieve greater control over the time‐to‐gelation, but is challenging to apply in deeply seated brain tissue cavities. Even though a fiber optic cable can be inserted, light distribution is unlikely to achieve a homogeneous gelation. In human patients, lesion cavities are larger, and more consideration needs to be given to the time to gelation/injection speed to ensure cavities are filled before gelation occurs.

### 3.2 Gelation of Bioscaffold to Conform to Tissue Defect Topology

In situ gelation is essential to retain the bioscaffold within the cavity. Although some pre‐gel is likely to permeate into brain tissue, sufficient material must be retained within the cavity to sustain cell invasion. A consistent interface between the bioscaffold and brain tissue is essential for cell invasion, as cells will not invade if this interface is not present.^[^ ^18^, ^38^ ^]^ ECM‐based hydrogel requires ≈50 min of curation to reach maximum gelation.^[^ ^42^ ^]^ Still, not all material is cross‐linked and provides a mobile fraction that can diffuse and permeate with surrounding brain tissue. Between 80% and 90% of material is gelled, depending on its protein concentration, which determines the level of cross‐linking of collagen fibers and bioscaffold stiffness.^[^ ^42^ ^]^ Stiffer UBM‐ECM (> 6 mg mL^−1^, G’ > 125 Pa) undergoes minimal degradation, whereas weaker hydrogel (< 4 mg mL^−1^, G’ < 75 Pa) disappears too fast to sustain cell invasion and the formation of *de novo* tissue.^[^ ^19^, ^38^, ^43^ ^]^ A 4 mg mL^−1^ UBM‐ECM protein concentration has been found to exhibit advantageous degradation characteristics, as well as tissue regeneration. MR elastrography measurements of human brain tissue indicate that this formulation is less stiff than mature brain tissue (G’ 1 to 3.5 kPa).^[^ ^44^ ^]^ This is consistent with the observation in wound healing, where a weaker granulation tissue supports cell invasion, whereas maturation of the tissue leads to stiffer characteristics. The rheological properties of bioscaffolds are therefore an essential design factor to achieve tissue regeneration.

The protein concentration of ECM hydrogels, as well as their rheological characteristics, are also determinants of their degradation. In addition to non‐gelled proteins, hydrolysis (i.e., degradation of cross‐linked proteins by their interaction with water) and erosion (i.e., washing out proteins from the hydrogel) degrade hydrogels. Stiffer hydrogels with a higher protein concentration in water degrade more slowly than weaker gels.^[^ ^42^ ^]^ For instance, an 8 mg mL^−1^ ECM hydrogel slowly and steadily degraded in water at a rate of 5% per day, whereas a 2 mg mL^−1^ formulation lost 70% of its proteins within 24 h, before undergoing a slow 2% daily decrease. The effects of hydrolysis and erosion are exacerbated with phosphate buffered saline (PBS), as well as artificial cerebral spinal fluid (aCSF), with a steady decrease of 5.7% per day (i.e., ≈80% of protein content is lost over 14 days) compared to distilled water (25% loss) for a 4 mg mL^−1^ protein concentration. However, hydrogel implantation occurs in a pathological environment, and proteases involved in lysing damaged tissues will additionally induce a biodegradation of bioscaffolds.

## 4 Impact of Pathological Environment on Bioscaffolds and Vice Versa

### 4.1 Proteolysis of ECM‐Based Bioscaffolds

**Figure 6:**
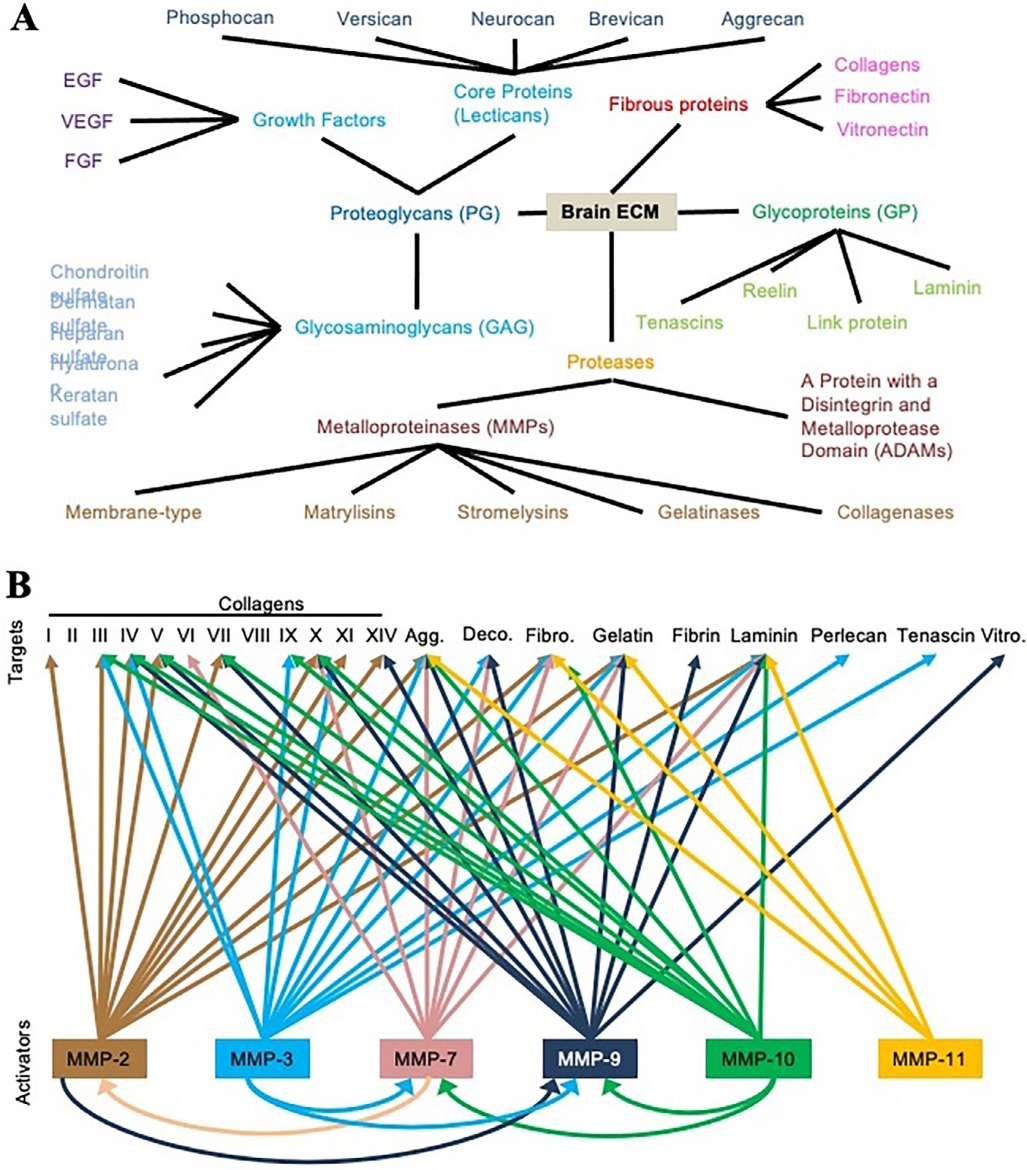
ECM and Proteases. A) Conceptualization of typical components found in the brain extracellular matrix (ECM). These components highlight the rich content that is present in decellularized bioscaffolds. B) Collagen in ECM bioscaffolds is considered the main structural component. Matrix metalloproteinases (MMPs) lyse different isoforms of collagen, but these same MMPs also cleave other ECM molecules. During this biodegradation process, signaling molecules, such as growth factors or juxtacrine factors, are released and influence cell invasion and differentiation.

**Figure 7:**
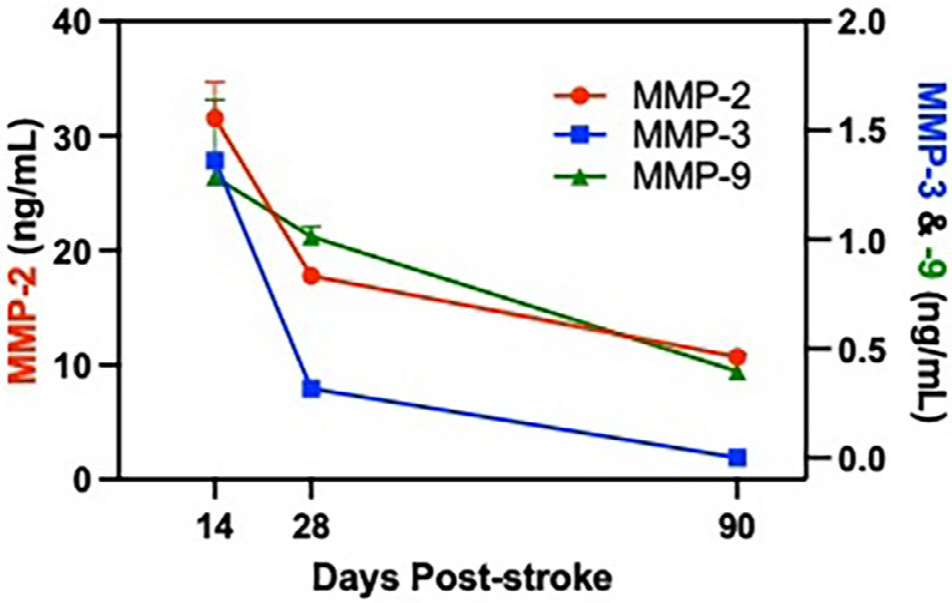
Matrix metalloproteinases (MMPs) in stroke‐induced tissue cavities. Extracellular fluid (ECF) evacuated during the implantation of ECM hydrogels at different post‐stroke intervals reveal MMP concentrations that act on these bioscaffolds. A continuous decrease of MMP‐2, ‐3, and ‐9 is observed between 14 and 90 days post‐stroke, highlighting the changing pathological context that these bioscaffolds encounter upon implantation.^[^ ^42^ ^]^ The lower MMP levels after 90 days will induce less biodegradation than the levels present at 14 days post‐stroke.

A variety of proteases are acting on tissues after acute brain injuries, as well as during neurodegeneration.^[^ ^45^ ^]^ The material composition of the injectable bioscaffold determines which proteases will cause biodegradation. ECM‐based hydrogels have a high collagen content that is cross‐linking during gelation. Proteases, notably matrix metalloproteinases (MMPs) within the collagenase family, are known to lyse these, as well as other proteins (**Figure** **6**). In stroke, MMPs are known to be upregulated and involved in tissue degradation. However, measurements of proteases are mostly restricted to the acute phase of tissue insults and are hence not providing an adequate description of the pathological environment in the tissue cavity. Evacuation of the ECF during the injection‐drainage process of bioscaffold injection provides an opportunity to measure protease in this environment.^[^ ^42^ ^]^ As pathological conditions evolve, the specific proteases, as well as their concentrations, will change. The pathological insult and age further affect proteolytic activities. During the acute phase, high levels of proteases are active, considering the widespread damage to cells and the clearance of ECM in the lesion core. However, as peri‐lesional tissue stabilizes, a return to tissue homeostasis is expected. As tissue cavitation occurs over a period of 7–14 days, the level of, for instance, MMPs is reduced (**Figure** **7**). Measuring MMPs levels in vivo is challenging, especially in the lesion cavity, and inhibitors typically affect multiple MMPs.

To specifically determine how, for instance, individual MMPs or their combination effect the biodegradation of injectable bioscaffolds, in vitro biodegradation studies can be used to investigate mechanisms of action under controllable conditions. In vitro studies using MMP‐2, ‐3, and ‐9, which are upregulated after stroke, demonstrate a dose‐dependent biodegradation of ECM hydrogel.^[^ ^42^ ^]^ However, the MMP concentrations within the cavity only add ≈20% additional degradation to the effects of hydrolysis over 14 days (70% degradation). Mixing of these MMPs at concentrations found in the lesion cavity further indicates that biodegradation is greater with a mixture of MMPs found at 14 days than 90 days post‐stroke,^[^ ^42^ ^]^ which is consistent with in vivo observations of biodegradation of implanted ECM hydrogels being greater at 14 than 28 or 90 days post‐stroke.^[^ ^19b^ ^]^ MMP‐9 exerted the most significant effect, with little added biodegradation occurring due to the addition of MMP‐2 and ‐3. However, MMP‐2, ‐3, and ‐9 individually exerted very similar levels of biodegradation by themselves.^[^ ^42^ ^]^ The in vitro biodegradation effect between 14 and 90 days MMP concentrations were only evident during the first week of biodegradation,^[^ ^42^ ^]^ potentially highlighting that certain cross‐links or proteins are more prone to biodegradation, whereas other cross‐linked proteins are more resistant or require a different set of proteases for lysis. In vivo mechanistic studies manipulating proteases and altering the biodegradation of ECM hydrogels are currently lacking. It hence remains unclear how the time course or rate of biodegradation affects tissue regeneration, although it is well established that a lack of biodegradation will prevent tissue regeneration and functional recovery.^[^ ^43^ ^]^

### 4.2 Permeation of Inductive Molecules into Brain Tissue

The degradation of bioscaffolds is influenced by their pathological environment, but molecules/proteins from the scaffold also permeate into the tissue surrounding the cavity. Glial scarring affects this process. During the sub‐acute phase, the scar is still forming and provides easier permeation of pre‐gel into surrounding tissue, whereas a mature scar provides a barrier to the bioscaffold/tissue interface and can contribute to less favorable outcomes.^[^ ^19b^ ^]^ Inductive bioscaffolds reduce scarring, and even with a mature glial scar, astrocytes can invade the scaffold and reduce the extent of scarring, hence gradually leading to its dissolvement.^[^ ^19a^ ^]^ Stiffer materials can lead to more extensive scarring and a foreign body response. Still, it remains unclear if the permeation of bioscaffolds into surrounding tissue is required to initiate the migration of cells into the scaffold or if other factors regulate this process. Hydrolysis and proteolysis lead to the release of bioactive molecules from bioscaffolds that affect peri‐implantation cells, as well as those that invade the hydrogels. ECM bioscaffolds contain growth factors naturally trapped within a tissue, such as nerve growth factor (NGF) and brain‐derived neurotrophic factor (BDNF), which are known to enhance neuronal survival and differentiation, as well as vascular endothelial growth factor‐A (VEGF‐A) associated with the induction of angiogenesis.^[^ ^46^ ^]^

ECM also contains a variety of cytokines, such as monocyte‐chemoattractant protein‐1 (MCP‐1/CCL2), which is thought to recruit peripheral macrophages and support their migration. Indeed, the infiltration of peripheral macrophages is considered a pivotal event in biodegradation. In the absence of macrophage infiltration, no biodegradation of the material occurs, and consequently, no tissue is regenerated.^[^ ^47^ ^]^ Fractionation of ECM hydrogels into structural and soluble components revealed distinctive roles of both involved in regulating macrophage phenotype. Soluble components that occur naturally in ECM shift macrophages from a pro‐inflammatory (M1) toward a pro‐repair (M2) phenotype through Notch and P13K/Akt signaling.^[^ ^46^ ^]^ The biodegradation of ECM bioscaffolds also releases matrix‐bound vesicles (MBV), which are essential signaling elements in tissues.^[^ ^48^ ^]^ The transfer of RNA, proteins, and enzymes occurs through these MBVs and promotes an immunomodulatory response toward a pro‐repair profile in cell phenotypes and cytokines.^[^ ^49^ ^]^ A major advantage of ECM bioscaffolds is their rich structural and soluble bioactive content that is reflecting the natural composition of tissues.^[^ ^50^ ^]^ However, the disadvantage is that it is difficult to modify or remove bioactive components from these materials. In contrast, bottom‐up engineered materials only contain bioactive content that is added during the production process.^[^ ^21a^ ^]^

### 4.3 Cell Recruitment and Formation of a De Novo Tissue

Scaffold degradation is an essential process to ensure that bioscaffolds release bioactive molecules, which recruit host cells to rebuild a new tissue. Within 6 h after implantation, immune cells invade the bioscaffold through the peri‐infarct tissue with a peak cell invasion ≈18 h post‐implantation.^[^ ^51^ ^]^ Immune cells lead the biodegradation process. The degradation of the scaffold aids the invasion of neural cells through the release of secreted chemokines, as well as juxtacrine factors (e.g., laminin), which provide motives for migration through an increasingly porous hydrogel. Within 24 h, host neural cells and endothelial cells (ECs) start invading, and by 14 days post‐stroke, most (60–90% depending on the timing of post‐stroke implantation) of the bioscaffold is degraded and replaced with *de novo* host tissue.^[^ ^18^, ^19^ ^]^ These host‐derived neural and vascular cells will secrete brain tissue‐specific ECM. The source of bioscaffold, hence, does not determine the cellular composition of the tissue being replaced. Ideally, the bioscaffold is only a temporary structure that supports the organ to reconstitute itself through its endogenous stem cells.^[^ ^18^ ^]^

There is an intricate interaction between NSCs and ECs that is driving the formation of tubules, leading to a re‐vascularization of the tissue.^[^ ^16^ ^]^ Both re‐vascularization and re‐neuralization need to occur side‐by‐side to ensure the viability of a newly forming tissue. As inductive bioscaffolds induce this endogenous cell response, neural and vascular cells are sourced from the surrounding peri‐cavity tissues. In these peri‐cavity tissues, an upregulation of cells occurs that is also increasing the number of cells in these damaged tissues.^[^ ^19b^ ^]^ Injection of liquid ECM hydrogel into the peri‐infarct area of stroke has been reported to induce behavioral recovery.^[^ ^52^ ^]^ Bioscaffolds can induce peri‐lesional changes that improve functional outcomes without regrowing new tissue. Bioscaffold implantation, therefore does not only promote tissue regeneration in a cavity, but also supports ongoing tissue repair in the surrounding damaged tissues.

## 5 From Small Rodents to Large Primates–Translational Challenges and Opportunities

### 5.1 Small Rodents – Proof‐of‐Principle Experiments

**Figure 8:**
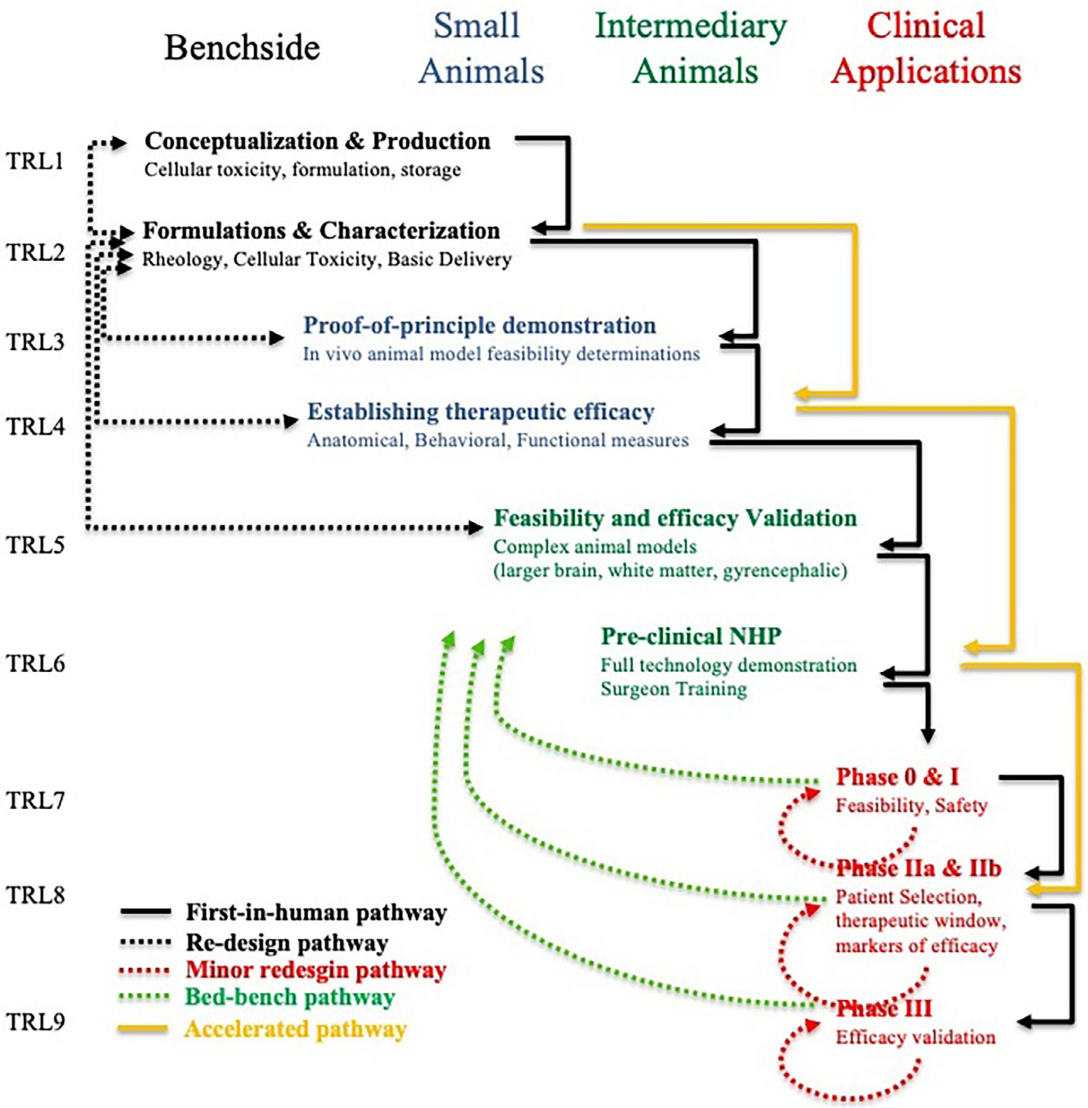
Translational pathway for bioscaffolds. Conceptualization of the translational pathway for injectable bioscaffolds to repair or regenerate brain tissue in relation to technology readiness level (TRL). A thorough first‐in‐human pathway is highlighted aimed at addressing the many technological barriers that exist in ensuring the efficacy of a bioscaffold. If a bioscaffold is not successful in each of these steps, either a complete re‐design is required or minor modification that would require re‐testing at the same TRL. Although ideally a linear path is desired, it is likely that many iterations of each step are required. It should also be expected that clinical trials can fail and that this does not necessarily invalidate the approach or material, but could be due to some technical aspects not being properly addressed in a previous stage. Once a bioscaffold has progressed in clinical trials and a re‐design or novel design is being tested, it is also conceivable that an accelerated pathway can be mapped out due to the redundancy of some technological questions (e.g., validation of image‐guided delivery).

Although injectable bioscaffolds for brain repair and regeneration are demonstrating feasibility and therapeutic potential in small rodent models of brain injury (e.g., mice, rats, and rabbits), the framework for the clinical translation from small rodents to primates, such as humans, requires further consideration (**Figure** **8**). Discovery and mechanistic investigations of how bioscaffolds can reverse pathological conditions are predominantly performed in small rodents.^[^ ^53^ ^]^ These animal models are best suited for initial studies of feasibility, such as the rapid screening of different types/sources of bioscaffolds, as well as establishing basic biological principles. The high reproductive rate of these animals, fast maturation rates, as well as space and cost efficiencies, are major advantages to economically develop promising candidates and eliminate ineffective designs. The homogeneity of subjects in these species conveys biological consistency, while a high level of experimental control provides typically standardized conditions to robustly evaluate biological effects using a small sample size. The favorable tissue healing properties and immune responses in rodents (especially younger subjects) further provide excellent testing conditions for putative tissue repair and regeneration strategies in mammals. Availability of transgenic mouse models is also important to investigate how particular physiological variables, such as macrophages, affect outcomes.^[^ ^54^ ^]^ A disadvantage of mice is their relative smaller size compared to rats and rabbits, which affects the bioscaffold delivery and can lead to an over‐estimation of regenerative effects in response to the permeation or diffusion of secreted molecules on host tissue.

To evaluate the maturity of technological development, Technology Readiness Levels (TRL, see **Table** **1**) are often used to describe the translational process (i.e., bench‐to‐bedside), whereas Manufacturing Readiness Levels (MRL) reflect progress in scaling‐up production and ensuring product quality (e.g., quality control and assurance procedures).^[^ ^55^ ^]^ TRL 1 covers the conceptualization of the approach and production of the bioscaffold, whereas TRL 2 would achieve the characterization of the material prior to testing in an animal model. Some studies might cover multiple TRLs. Small rodent investigations would typically cover TRL 3 and 4, which pertain to demonstrate feasibility experimentally in a model system and establish proof of function, i.e., therapeutic efficacy. Once the basic proof‐of‐principle has been established in small rodent models, these principles need to be evaluated in models that are replicating more complex features (i.e., larger tissue defect, more complex anatomical organization).

**Table 1:** Technology Readiness Levels (TRLs) for injectable bioscaffold to repair/regenerate brain tissue. NHP = non‐human primates.

TRL	State of Development	Species
1	Conceptualization and Production of Bioscaffolds (e.g., material sourcing, defining a production procedure, reproducibility and batch variation)	*In silico, ex vivo, in vitro* (mouse and human cells)
2	Formulation of bioscaffolds and their characterization (e.g., characterization of biomaterial composition, biomechanical properties, gelation characteristics, cytocompatibility, injectability)	
3	Experimental proof of principle in animal models–feasibility (e.g., defining implantation procedure and timing of administration, biocompatibility, cellular response, encapsulation/foreign body response, tissue restoration)	Mice, Rats, and Rabbits
4	Validation in animal models–therapeutic efficacy (e.g., histological characterization of tissue repair/regeneration, functional brain changes, behaviral recovery)	
5	Feasibility and efficacy validation in more complex animal models (e.g., large tissue defects, gyrencephalic brains)	Ferrets, Dogs, and Sheep
6	Full technological demonstration of therapeutic efficacy in pre‐clinical setting (e.g., large tissue defects and time course of tissue regeneration requirements for adjunct therapies such as physical therapy, image‐guided implantation, long‐term safety and maintenance of efficacy, thorough evaluations of risks for tumor formation and seizures, identification of potential adverse effects in clinical trials, training of surgical team)	Large NHP
7	Phases 0 and 1 clinical trial (e.g., patient recruitment, inclusion/exclusion criteria, technical feasibility and safety in chronic human patients)	Human
8	Phase 2 clinical trial (e.g., patient selection, narrowing of therapeutic window, dosing, identification of markers of efficacy, selection of primary endpoints, determination of time points of assessment and long‐term surveillance)	
9	Phase 3 clinical trial–full technical demonstration in target population (e.g., efficacy validation, comparison to standard of care)	

### 5.2 Importance of Intermediate and Large Animal Models to Demonstrate Technology Readiness

Although there is a high conservation of biology between small rodents and large primates, size, anatomical and physiological differences can impact outcomes and interpretation of effects.^[^ ^53^ ^]^ Primates have larger brains and exhibit a more complex tissue structure. For instance, rodents have lissencephalic brains (i.e., the cortex lacks gyri and sulci), whereas humans have gyrencephalic brains. Intermediary species, such as ferrets or sheep, can be used to establish how gyrencephalic brains could be repaired/regenerated using bioscaffolds.^[^ ^56^ ^]^ These studies would be considered to fall within the TRL 4–6. In primates, white matter constitutes a greater part of the brain and is severely affected in acute brain injuries, including Wallerian degeneration that affects areas remote from the primary insult. It currently remains unclear how bioscaffolds would impact white matter tract recovery. The large volume of most primate brains also creates new delivery challenges in terms of image‐guided surgical implantation, as well as the volume to be delivered. The large bioscaffold volumes require a scaled‐up production and address additional biological questions, such as if there is a maximum cavity volume that can be replaced through endogenous cell invasion.

Considering that primates are less efficient at tissue healing, these questions might require the development of more auxiliary approaches (e.g., growth factor delivery) to ensure an efficient tissue regeneration. The longer life span of intermediary species and primates is also better suited to determine the long‐term effects of these injectable bioscaffolds and how aged, rather than young, subjects will respond to these materials. By using non‐human primate (NHP) models, researchers can obtain more accurate and predictive data on the effectiveness and safety of bioscaffolds before testing them in human clinical trials. NHP models can address key clinical parameters in pre‐clinical settings at TRL 6–7. Especially the evaluation of biomarkers, such as neuroimaging of a variable brain anatomy, assessment of bioscaffolds’ impact on complex behaviors (e.g., fine finger movement), and the impact of rehabilitation strategies, needs to be established in appropriate NHP models.^[^ ^57^ ^]^ Clinician training and long‐term outcomes of bioscaffold implantation, including potential immune responses, tissue integration, and functional recovery, need to be evaluated in NHP models prior to authorization of first‐in‐human trials.

### 5.3 Considerations for Clinical Trials

First‐in‐human trials of bioscaffolds to regenerate brain tissue are likely to consist of technical feasibility trials (sometimes referred to as Phase 0) in small cohorts of patients (n <10). These initial trials could ensure that the technology (bioscaffold, surgical tools, cranioplasty, duraplasty, biomarkers) is ready to assess safety in a Phase I clinical trial (n >10, TRL 7). In the case of injectable bioscaffolds, this will involve the safety of the material itself, as well as establishing if any ill‐effects are caused by the implantation surgery. Some biomaterials, such as ECM‐based bioscaffolds, have been used extensively in patients without major safety concerns, but these were mostly outside the brain or for duraplasty.^[^ ^58^ ^]^ However, most of these applications were sheets of ECM, rather than hydrogel preparations for volumetric tissue regeneration.

Phases 0 and I could be conducted in chronic patients, which fall outside the therapeutic window established in animal models. Unlike drug studies, these materials cannot be administered to healthy controls, and only subjects with appropriate pathological conditions can be included. It provides a means to rapidly advance methodology without compromising efficacy testing. This approach was used in the initial translation efforts for cell implantation to treat neurological disease.^[^ ^59^ ^]^ Cell and gene therapy Phase I trials for neurological conditions have repeatedly demonstrated that stereotactic surgery in itself is safe,^[^ ^60^ ^]^ but demonstration of therapeutic efficacy has been more challenging.

Filling of cavities through an injection‐drainage approach has not been performed in human patients. Image‐guidance will be an important aspect of these trials.^[^ ^61^ ^]^ A major advantage in human subjects is the availability of intra‐operative MRI, which affords monitoring of tissue changes during the procedure.^[^ ^62^ ^]^ The development of interventional and diagnostic imaging in patients will considerably improve the neurosurgeon's ability to deliver and monitor bioscaffolds and their impact on tissue repair and regeneration. The identification and validation of biomarkers based on neuroimaging would be an important aspect of Phase II clinical trials (TRL 8) aimed at establishing an appropriate dosing strategy and therapeutic window (Phase IIa), as well as markers of efficacy (Phase IIb). Phase II will provide crucial data about patient selection, effect sizes, and subject variability, which is required to calculate the number of subjects needed to enroll in a therapeutic efficacy Phase III trial (TRL9). Phase III will recruit a large number of subjects and might require placebo‐controls.^[^ ^63^ ^]^ Considering that the implantation of bioscaffolds involves a neurosurgical intervention and extensive diagnostic imaging, these will be costly undertakings. Phase IV will consist of market surveillance, which has been shown to be essential for identifying the ill‐effects of bioscaffolds (e.g., surgical meshes) in some conditions.^[^ ^64^ ^]^

We here follow a similar TRL assignment than suggested for bioprinting, with TRL 7–9 corresponding to Phases I, II, and III clinical trials.^[^ ^65^ ^]^ However, the conceptualizations of TRL for gene therapy considered a TRL9 to be post‐approval commercial deployment.^[^ ^66^ ^]^ Depending on the technology, some differences in categorization can be envisaged. The development of new surgical tools required to deliver bioscaffolds by themselves, for instance, might be conceptualized on a separate tangential TRL scale, which would converge with Phase 0 or I surgical trials.

## 6 Conclusion

The translational journey of bioscaffolds from small rodents to large primates is a complex and multifaceted process that involves addressing challenges and leveraging opportunities. Injectable bioscaffolds represent a promising avenue for the repair and regeneration of brain tissue. While significant progress has been made in understanding their design, delivery, and integration, several translational challenges remain. An increasing shift toward in vivo investigations is required to determine the technical and biological conditions that support successful therapeutic effects. Although it is tempting to assume that merely improving the design of materials is sufficient for therapeutic efficacy, addressing these translational challenges through interdisciplinary research and collaboration will be essential to realize the full potential of injectable bioscaffolds in clinical applications for brain tissue repair/regeneration. By carefully navigating these challenges and capitalizing on the unique advantages of small rodents, intermediate and NHP models, researchers and clinicians can jointly advance the field of regenerative medicine and ultimately improve the quality of life for patients with acute brain injuries.

## Conflict of Interest

The authors declare no conflict of interest.

## Author Contributions

M.M. conceived of and drafted the manuscript. A.K. edited the manuscript.
